# Exploring the role of external experts in supporting staff to implement psychosocial interventions in care home settings: results from the process evaluation of a randomized controlled trial

**DOI:** 10.1186/s12913-019-4662-4

**Published:** 2019-11-04

**Authors:** Claire A. Surr, Emily Shoesmith, Alys W. Griffiths, Rachael Kelley, Joanne McDermid, Jane Fossey

**Affiliations:** 1 Centre for Dementia Research, School of Health and Community Studies, Leeds Beckett University, Leeds, LS1 3HE UK; 2 Kings College London, London, UK; 3 Oxford Health NHS Foundation Trust, Oxford, UK

**Keywords:** Care homes, Dementia care mapping, External expert, Intervention implementation, Long-term care, Practice development, Process evaluation, Psychosocial interventions, Qualitative

## Abstract

**Background:**

Psychosocial interventions offer opportunities to improve care for people with dementia in care homes. However, implementation is often led by staff who are not well prepared for the role. Some interventions use external experts to support staff. However little is known about external expert, care home staff and manager perceptions of such support. This paper addresses this gap.

**Methods:**

Multi-methods study within a process evaluation of a cluster randomised controlled trial of Dementia Care Mapping™ (DCM). Interviews were conducted with six external experts who also completed questionnaires, 17 care home managers and 25 care home staff responsible for DCM implementation. Data were analysed using descriptive statistics and template analysis.

**Results:**

Three themes were identified: the need for expert support, practicalities of support and broader impacts of providing support. Expert support was vital for successful DCM implementation, although the five-days provided was felt to be insufficient. Some homes felt the support was inflexible and did not consider their individual needs. Practical challenges of experts being located at a geographical distance from the care homes, limited when and how support was available. Experts gained knowledge they were able to then apply in delivering DCM training. Experts were not able to accurately predict which homes would be able to implement DCM independently in future cycles.

**Conclusions:**

An external expert may form a key component of successful implementation of psychosocial interventions in care home settings. Future research should explore optimal use of the expert role.

## Background

Care homes make an important contribution to care for people with dementia. Over a third of people with dementia live in such settings [1], equating to 50–70% of the care home population [1, 2]. Ensuring they provide good person-centred care for people with dementia is an international priority [3] and psychosocial interventions can support this, with care home staff often leading or supporting implementation [4]. However, staff may not be adequately prepared to undertake this role, given the low levels of education, training [5], literacy and numeracy among the workforce [6, 7]. Annual turnover rates of 30–50% [8] and inadequate staffing levels [9] can lead to staff burnout and poor morale [10], which impact on capacity and motivation to implement interventions.

Many trials of psychosocial interventions lack thorough implementation evaluation. However, a range of common barriers and facilitators have been identified [11, 12]. They include: staff motivation, attitudes and confidence to implement the intervention; use of top-down implementation approaches reducing staff/team ownership of the intervention; time, competing priorities and staff turnover; the degree of management support; whether specific support for intervention implementation is provided (e.g. supervision, mentorship); staff perceptions of whether the intervention is practicable and will improve care/resident well-being; and whether there is a ‘learning organisation’ culture. While use of top-down implementation can, therefore, be a barrier to implementation, it may be facilitated by provision of appropriate ongoing support from an expert who is external to the service setting. The expert may be a member of the research team, or an independent expert in use/application of the intervention provided/funded by the study, but who is not a member of the immediate research team.

External expert support has been employed in a number of care home trials with good success. Fossey et al. [13] used expert-led skills modelling and staff supervision alongside staff training to successfully reduce neuroleptic medication use. Edwards et al. [14] combined staff training and an action planning toolkit with ongoing implementation support from external expert. Staff reported the support facilitated their ability to implement practice change and the researchers felt this role was pivotal to intervention success. Likewise, support from an expert was crucial for successful implementation of an end-of-life care programme in nursing homes [15].

However, despite the reported successes of utilising external support, little research has examined the role from the perspective of external experts or care home staff. Fossey and colleagues examined external expert experiences [16] and separately staff experiences [17], of receiving weekly support from the expert. The experts identified the gradual process of building relationships with each home and its staff as important in engaging them with the intervention. Enabling care home staff to see direct benefits of implementing the intervention supported ongoing motivation. The experts felt they were important agents for change through delivery of effective training and supporting staff to reflect on their practice. Care home staff valued the consistent presence of the external expert throughout the trial, which enabled the expert to engage with specific issues faced in each home. Staff felt supported through the expert working alongside them, rather than instructing them.

### Dementia Care Mapping

Dementia Care Mapping™ (DCM) [18] is a psychosocial intervention used for over 20 years within care home settings [19]. It is an observational tool set within a practice development process, aimed to assist person-centred care delivery. DCM is founded on the basis of staff having an underlying knowledge of person-centred care and provides them with evidence, from the day-to-day experiences of care home residents, of whether person-centred care is being delivered. It also supports identification of good and poor practice and the development of practical action plans, to continually improve care for individual residents and the setting as a whole. The standard model of implementation in the UK is as follows. Following training, two staff members lead DCM cycles, which include 1) briefing staff about DCM, 2) observing and recording resident’s care experiences, 3) analysing the data, 4) preparing a feedback report and holding feedback sessions and 5) creating individual and care home level action plans [20]. Unlike many psychosocial interventions, DCM has the advantage of international use in care home settings over a sustained period [19]. A small number of DCM trials have produced mixed results. Studies using researcher led DCM cycles have demonstrated resident level benefits [21–23] while those with care home staff led cycles have not [24, 25]. However, externally led DCM cycles are not usual practice. Anecdotal evidence from practice suggests it is however, feasible for care homes to implement DCM successfully [26–28]. There remains limited robust evidence evaluating the features of successful DCM implementation [29]. As with other care home interventions, common challenges include the time requirements for training, mapping, feeding back and implementing changes [21, 30]; workload and staffing pressures [23, 31]; trained staff (‘mappers’) not feeling adequately prepared to implement DCM [32]; and lack of organisational and/or management support [21, 25, 31].

### The DCM EPIC trial

**Fig. 1:**
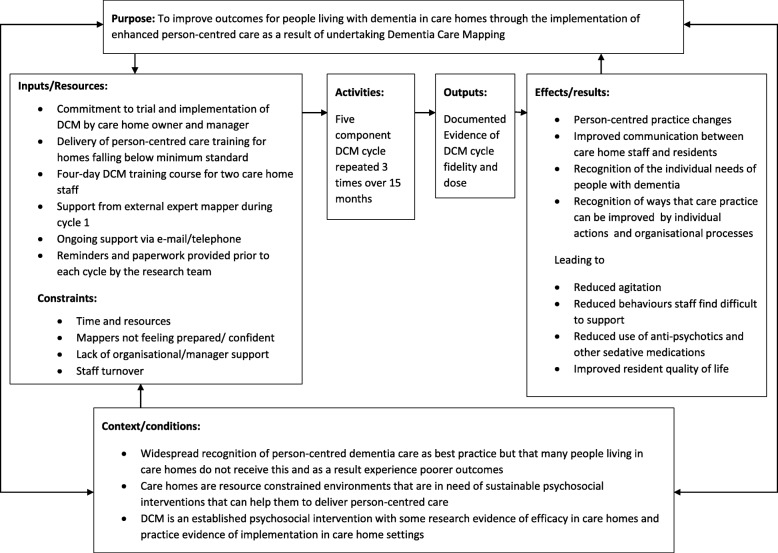
Logic model for DCM

The DCM EPIC cluster randomised controlled trial was a pragmatic, definitive trial of the effectiveness and cost effectiveness of DCM in care home settings [33, 34], including a full process evaluation. Care homes were initially screened for provision of dementia awareness and person-centred care training to staff and additional training was provided where homes fell below a minimum in terms of content and reach across the staff group [35]. Two staff from each intervention care home were trained to use DCM and asked to complete three DCM cycles (3-months, 8-months and 13-months post randomisation) using standard procedures [20]. Each cycle included: holding at least one briefing session; observing up to five residents with dementia (depending on confidence and skill) for up to 6 hrs over a single week; analysing the data and producing a standardised feedback report; delivering at least one formal feedback session; and producing action plans for each resident observed and one for the whole home/unit. A logic model for implementation is provided in Fig. 1.

In accordance with the logic model while adopting a pragmatic design, feasible approaches to support DCM implementation that were not part of usual DCM practice were adopted, including support from an external expert (expert mapper). Rather than expert led implementation as in previous trials [22, 23], the expert supported mappers during their first DCM cycle. Ongoing advice and support was then available by phone/e-mail if needed. Experts assessed mapper capability across the cycle components. Understanding whether variable degrees of external support could be provided depending on care home and mapper needs is of interest for future cost-efficient implementation, if experts can accurately judge the level and length of further mappers may need to successfully implement DCM.

## Methods

This paper aimed to answer the following questions:

1. What were expert mapper, mapper and manager perceptions of the role expert mappers played in supporting DCM implementation?
2. What DCM implementation issues and challenges were present?
3. Were expert mappers able to accurately identify which care homes would have ongoing DCM support needs?

A multi-methods process evaluation drawing on qualitative and quantitative data from a number of sources, following the Medical Research Council guidelines [36] on process evaluations was conducted. The MRC guidance outlines three core components to a process evaluation: implementation, mechanisms of impact and context. Implementation is concerned with the ‘what’ and ‘how’ of implementation. What was implemented in this trial has been reported elsewhere [37]. With regard to mechanisms of impact, the intervention failed to demonstrate any measurable changes on the primary or secondary outcomes [38], although some perceived benefits and potential mechanisms of impact were identified [38, 39]. This paper, in exploring the role of the external expert mapper, reports on the ‘how’ of implementation and identifies some of the contextual issues associated with implementation.

### Setting

Fifty care homes were recruited to the trial, from three regions of England with 31 randomised to DCM. Quantitative data on intervention fidelity was collected in all intervention homes. Qualitative process evaluation data was collected in a sample of 18 of the DCM intervention homes, representing the variability of DCM implementation across the trial (0–3 cycles). Selection for participation in the qualitative process evaluation was undertaken by the trial manager, based on assessment of data returned on number of completed DCM cycles throughout the trial period.

### Participants

Participants were expert mappers (experienced DCM users provided by the research team), care home managers and care home mappers.

### The expert mapper role

Seven expert mappers took part in the trial. They were recruited from the University of Bradford’s existing network of DCM Trainers and Evaluators. There are four levels of DCM training/expertise (Basic User, Advanced User, Evaluator and Trainer) which include a combination of face-to-face theoretical and practical training, gaining DCM experience in practice, and rigorous assessment. DCM Evaluator status is no longer available within the University of Bradford DCM training portfolio. However, existing DCM Evaluators are qualified to undertake full DCM cycles in organisations external to their own on a consultancy basis. To qualify they needed to have completed Basic and Advanced User training, at least 60 hrs of DCM observations as part of full DCM cycles and in a range of settings, and an extensive assessed report on DCM implementation. DCM Trainers are those qualified to deliver the standard four-day DCM course to others. They need to have previously completed DCM Evaluator status and to have delivered at least four assessed DCM Training courses. DCM Trainers and Evaluators are therefore, highly experienced in use of the DCM tool and process. Six of the experts in this study were DCM Trainers and one an Evaluator. All were qualified health professionals (nurses, occupational therapists, speech and language therapists), currently or previously working in health and social dementia care services. All had been qualified to Trainer/Evaluator level for at least 2 years and most for significantly longer (10+ years), conducted many DCM cycles in their own and external organisations, undertaken supervision of newly qualified and experienced mappers, and co-ordinated programmes of DCM within their employing organisation. They were therefore highly experienced in implementation of DCM and in mentorship and supervision of newly qualified mappers. A 1 day training on the trial protocol, processes, and use of the standardised trial documentation was provided to all experts prior to them commencing the role. Experts were introduced to the care home mappers and managers by the DCM lead for the trial, or the trial manager, after randomisation. Some mappers were trained in DCM by the expert who supported them.

### Care home mappers

Care home mappers were identified by the home manager in discussion with a researcher using a set of criteria/qualities required, developed by the research team. These included being able to speak and write English well enough to undertake the training and produce a DCM report, having IT skills commensurate with producing a simple report, having enthusiasm for the role etc. The full criteria are reported elsewhere [38]. They also needed to be able and willing to attend a four-day DCM training course.

### Data collection

Following completion of 16-month follow-up data collection, face-to-face or telephone semi-structured interviews with the expert mappers and face-to-face interviews with the mappers and managers were conducted by a researcher, using a topic guide developed by the research team (see appendix 1). The interviews were conducted by a total of 11 researchers. Most were female (8; 73%) and their average age was 24. These were audio recorded and transcribed verbatim. Mapper (individual or pairs) and manager (individual) interviews took place in the care home in a private location. Expert mapper interviews (individual and one conducted as a small group *n* = 3) took place by telephone or in person in a private location in their workplace. Researchers were independent of the DCM intervention. Interviews focussed on experiences of implementing DCM and the expert mapper support. Expert mappers completed a standardised, data collection form following completion of the supported mapping cycle. It contained closed and open questions about DCM cycle component completion (briefing, mapping, analysis, feedback and report writing, action planning), the degree of support required, whether they were confident in mapper’(s) ability to accurately undertake the component in future cycles (confident, somewhat confident, somewhat unconfident, unconfident) and whether they judged mappers would need further support with a component in the future (Yes/No). All interview participants were approached in person by the researcher and received an information sheet detailing the purpose of the interviews and were provided with the opportunity to discuss the study with the researcher ahead of participation. Care home staff were approached initially by the care home manager and details of staff willing to participate were provided to the researchers. Written consent was gained from all participants.

### Data analysis

Interview data was analysed using template analysis [40]; a form of thematic analysis, by the research team. Eleven researchers were involved in data analysis. Each transcript was independently coded by two researchers; one who had collected data in that home and one who had not. After coding an initial set of 11 transcripts the research team discussed initial themes and developed a coding template. This comprised a coding tree made up of higher level codes (e.g. manager influence, barriers and facilitators, mappers) each with a number of sub-codes (e.g. DCM ownership, change of focus/priorities, resources, expectations vs reality, mapper confidence). The remaining transcripts were independently coded by two researchers from the team, after which each pair discussed their analysis and agreed coding within the template framework. Development of the coding template continued throughout data analysis, informed by the developing themes and team discussion of analytic interpretations, including comparison of codes and themes across care homes and between different types of participants. To support coding via multiple researchers, coding was completed using tables in Microsoft Word. Data from the expert mapper forms were analysed using thematic analysis and descriptive statistics. Data from the different sources related to the expert mapper role were then brought together to identify the main findings presented.

## Results

Seven expert mappers supported at least one care home to complete a cycle. Six experts took part in an interview; three individual and three in a small group. One expert retired before the end of the trial. Expert data collection forms were returned for the 28/31 intervention homes that had completed at least one component of 1 cycle. Three homes did not complete any DCM components during cycle one and so the experts were unable to provide data for these homes. Interviews were undertaken with 17 care home managers and 25 care home mappers. Two managers were also care home mappers. Interviews ranged from 5 to 38 min in length for managers and mappers and 31–92 min for expert mappers. Quotations are anonymised and identified by the role of the speaker. I for interviewer, EM for expert mapper, CH for care home identifier and the speaker role.

### Experiences and perceptions of the expert mapper role

Interviews with expert mappers, care home mappers and mangers led to three major themes: the need for expert support; practicalities of support; and broader impacts of support. Each contained a number of sub-themes.

#### The need for expert support

There was universal agreement across participants from all three roles that the input of the expert mapper was needed and valued by the care home mappers and care home.

*I: How do you think the trial would’ve worked differently without your support?*

*EM70002: I can’t see it would’ve happened. .. Would you?*

*EM70003: No, not at all (Small group interview with EMs)*

*She helped us a lot, because when we started to do it I couldn’t understand what we are doing. (Mapper CH58747)*

*When she’d gone the support had gone … (Mapper CH50010)*

Despite such support not being provided as standard following DCM training, it was viewed as essential in implementing DCM.

*I think [without expert mappers] you would’ve had more people not map, and probably lower quality data. (EM70006)*

*We had support from the other mapping lady [expert mapper]. She helped us underway with that. But the second time round we didn’t, we didn’t get that far. (Mapper CH50010)*

*I feel that the mappers will require long term support in order to assist them in producing work in the agreed schedule and help with English written skills* (Cycle one report: EM2)

**Fig. 2:**
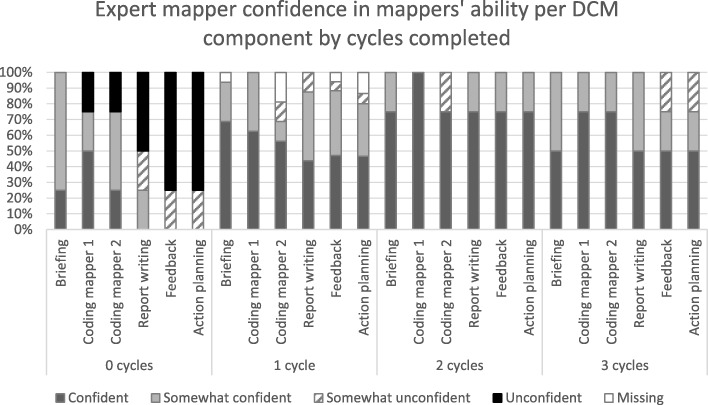
Expert mapper confidence in mappers’ ability per DCM component, by actual cycles completed

Figure 2 shows how confident the expert mappers were, in care home mapper’s abilities to use each DCM component accurately in future cycles, after the supported cycle one. This is broken down by cycle component, and in relation to the mapping component, by each mapper’s ability to use the DCM coding frames that comprise the ‘mapping’ activity accurately. This is presented with the care homes grouped by whether they subsequently completed none, one, two or 3 cycles. The three care homes who did not complete any DCM components are not included in the data. The figure shows that while expert confidence increased across the DCM components for homes where one or more cycles were completed compared to none, there remained a lack of confidence generally in mappers’ abilities to use DCM in the future. The experts were confident or somewhat confident in mappers across all DCM components in only 15 out of the 28 (53.6%) homes.

**Fig. 3:**
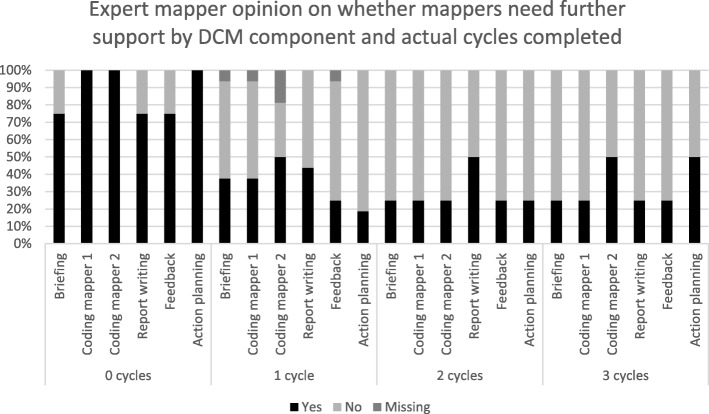
Expert mapper opinion on whether care home mappers need future support on each DCM component by actual cycles completed

Figure 3 shows the expert’s perceptions of whether mappers would benefit from support with each DCM component in future cycles. This is presented grouped by actual cycle completion. While further in-person support was not available in the trial, the experts perceived that mappers across 18 of the 28 homes (64.3%) would benefit from additional support for at least one DCM component in future cycles.

Sub-themes on the nature of external support included: the experts’ contribution; types of support; ensuring a correct balance; and recognising boundaries.

The expert mappers’ contributions covered several areas including ensuring accurate use of DCM, helping to avoid or rectify issues when DCM was incorrectly implemented and support for the development of mapper skills and confidence.

*I think some of the classic mistakes that can be made in DCM would’ve been made, … And if they hadn’t been picked up and supported or changed, it can have a really devastating effect on DCM. (EM70002–4)*

*I felt I struggled when [expert mapper] went. It’s like when she was there it’s “yeah I’ve got this, I know this, she refreshed me”, but, and then it was like actually I don’t feel that confident with it (Mapper CH50028)*

*She said it was one of the highest [Inter-rater reliability] scores she’d seen … . So it gave us confidence to know we were both on the same wavelength, and we both knew what we were doing. (Mapper CH50031)*

Expert mappers in particular identified how a number of the care home mappers were not able to accurately apply DCM and thus their input was vital in ensuring, for example, that feedback and action planning were based on accurately coded data.

*… we need to support them with the IRR. The inter-rater reliability. So they know that, do you know what the coding, getting the coding right for different things. (EM70005)*

However, this type of support was not always valued by the care home mappers. They did not necessarily appreciate being advised about how to work with residents they felt they knew well and the expert mapper did not.

*I found it a bit constricting really [laughs] … she’s mapping at the same time as you and she’s picking up different things that you are, … and I at the end of that first one, we were a bit, little bit demoralised. Because we picked up on certain stuff and she’d seen something else differently. So you think ahh! I’ve done that wrong, or you know why didn’t I see? … you kind of wanted to say you know well that’s, … I know that resident. You know you’re only here for the day … (Manager/mapper CH50069)*

*I think we found that she was picking up on things, maybe a little too picky. I don't know how to describe it - things that we knew why they were happening, and that, you know what I mean, she was making big issues out of them even though we sort of knew, this is normal for this person. (Mapper CH58930)*

In other cases, experts felt their input was vital to keeping DCM implementation on track when things went wrong.

*… when it came to feedback, erm, I did much more in that feedback session than I, I probably should have done, but I was trying to salvage a situation that was going terribly wrong. (EM70006)*

*"Extensive support required in writing the DCM report due to English written skills. Both mappers had been unable to complete the report by the required time schedule" (Cycle one report: EM2)*

Thus perceptions differed about the need for and value of the expert input that could support or inhibit implementation.

The types of expert support provided included advice and encouragement through to, in some cases, taking over from mappers to ensure that materials or processes were completed or accurately implemented.

*… but I did need to say “try and make it easy on yourself, be really well prepared”. (EM70002–4)*

*… two of the homes I worked with where they were quite slow at sending me the documents, it’s because they weren’t confident on the computer. So I had to basically rewrite it for them ... So although they had the template, it was kind of a big thing for them to actually go on the computer and do it. (EM70006)*

Ensuring the correct balance of support was seen as important. Experts felt the pressures of trying to support care homes to implement DCM in line with the standards and schedules of the trial, whilst also maintaining positive and supportive relationships with the mappers.

*I felt like a sort of jobbing rep trying to get their attention, rather than making sure they were equipped to participate in this fantastic trial. You know and I think that’s what happens in care homes, is that external sources become a nuisance really. (EM 70002–4)*

*.. it’s been very challenging to, to word things in the right way which don’t lead to one word answers or to people feeling and knowing that they haven’t done what they needed to do and me putting pressure on them in the nicest possible way (EM 70002-4)*

However, from the perspective of mappers this balance was not always achieved.

*I kept getting e-mails and various things, "Why isn't this done? This needs doing now, this needs doing", and it was quite stressful as well because most of that, I was doing on my own. (Mapper CH58930)*

The boundaries of expert mapper support were at times unclear, with experts sometimes involved in supporting relationships and key staff engagement that would be necessary to facilitate DCM implementation.

*With two of the homes I worked in, ... their relationship with the manager wasn’t always an easy one and there was lots of “could you talk to her about what we need to be doing? … . I asked that she didn’t attend the feedback session … and I don’t know whether that was the right thing to do or not. But I said I felt that perhaps staff would be, feel more able to contribute without the manager being present. (EM70006)*

#### Practicalities of support

A range of practical issues associated with provision of expert mapper support were identified. These included: communication; proximity; time available; relationships; and flexibility.

Two closely related sub-themes were the importance of good communication between the expert and the care home mappers, and the impact of experts’ proximity from the care home.

Communication with the care home managers and mappers could be challenging and difficult to maintain for experts. A reliance on telephone and e-mail, meant experts had to call when the appropriate person was on shift and available, or e-mail. This latter method was often ineffective if mappers did not have work e-mail addresses or checked and answered them irregularly.

*So I’m ringing a home in [location] saying can I speak to … and then you get someone who doesn’t know who that person is. You know so it just felt like communication links were really tenuous. (EM 70002–4)*

*I personally prefer some person who’s come in here. Emailing and Skypeing is a bit different because physically I mean she could have helped us with our computer. (Mapper CH58747)*

A small number of expert mappers were utilised during the trial, to maintain consistency in support and because of the small number of individuals with the requisite experience to take on the role. The experts were DCM trainers or had led organisational programmes of mapping and supervised teams of mappers over many years. This, however, meant some experts had to travel large distances to support care homes.

Expert proximity to the care home caused challenges through requirements to work long days and could preclude last minute changes to arrangements or working flexibly with mappers across a cycle.

*… if she was a local person who could actually pop in and say look I’ll come to the end of your mapping, or I’ll come to the right beginning of the mapping and see how you get on. That would have made a huge difference to them, because they would have felt like okay I can give her a ring and she can come in for a bit. (Manager CH50011)*

*She travelled quite far so she had to leave at a certain time, and I feel that if I’d had her a bit longer or an extra day that I would’ve felt more confident (Mapper CH50028)*

Expert mappers felt this could have been addressed by condensing the mapping cycle into a single intensive week.

*I was too far away. The day and one home that I’d stayed the night, you know gone down by train, stayed the night, arrived. … and just thinking … I wish I was moving in for the week, you know, then we could timetable it all without it being too big a challenge. (EM70004)*

However, for the care home mappers and managers the cycle being spread over a month, already felt intensive and difficult to support.

*… she [the expert mapper] was a little unrealistic about what was, you know, the routine of a nursing home and the fact that the mappers were also carers and nurses and had other you know, activities and tasks and jobs to do (Manager CH58930)*

*The time before [cycle 1], when I was trying to cram it all in to this time period that I kept getting told, "It had to be this, it had to be, it had to be". I ended up being off ill by the time I'd finished because I was just so shattered … The last couple of times, I've spread it out a little bit more and haven't had as much aggro. It is still taking a lot of time, but I haven't felt as pressured (Mapper CH59830)*

The amount of time available for support (5 days of expert time) was consistently felt not to be enough to adequately support sustainable DCM implementation.

*It just didn’t seem long enough as an expert, you know, to rush it through. I just felt I could spend days with these people. (EM70003)*

*It probably would have been more helpful if perhaps they [expert mapper] … had visited the home. Perhaps once or twice during the programme, ... I feel as if we were, had the training and then left to our own devices really. (Manager CH50024)*

*But the time we didn’t have the support like [cycle 2], when we do observation ourselves. So the difficult, the difficult time it was when we didn’t know what to do, how to put the coding on, so we just try to decide together, with my colleague. … That was difficult for me. (Mapper CH58747)*

Building a positive relationship with the care home mappers was viewed as crucial to successful engagement by the experts.

*There was a lovely lady that come and marked me, we done mapping together. I think her name was [expert mapper name]. She come and that was brilliant. (Mapper CH50028)*

*She [Expert mapper] was brilliant. … the one we had really lovely lady. She was like, she explained everything to us, you know, because she watched us. And it was really good actually because we felt quite special. (Mapper CH12792)*

Flexibility of the expert mappers to work with the care homes and mappers needs, as well as to work within the trials processes was supportive of implementation.

*She was really, really good, she was very, very friendly, she was very understandable. Each time when we were not able to facilitate, accommodate the staff with her time-table, she was really understandable. She was then coming after five o’clock. Coming spending time with the staff. (Manager CH50028)*

However, where experts believed there to be specific trial requirements that could not be approached flexibly (actual or perceived) this led to them feeling pressurised and uncomfortable. It might also mean mappers were pressurised to implement DCM in a way that was not feasible for them to achieve.

*And I think to expect them to map multiple people for a number of hours, it created a lot of anxiety for people, and it went against me as a DCM trainer, as an expert mapper … I would never ask that person generally to go on and map multiple people. But I kind of felt that pressure ... (EM70006)*

*The expert mapper was a little full on. Knew her subject, very passionate, erm, but very, erm, time scale orientated, which kind of pushed, I think added to the stress. (Manager CH58930)*

#### Broader impacts of the expert role

Two broader impacts of involving expert mappers were identified: experts’ personal development and DCM development. All expert mappers identified learning they had acquired from taking on the role. This included skills in working with organisations to nurture DCM and reminders of the challenges faced when first implementing DCM.

*I felt very privileged to be part of it, but I also found it one of the most challenging things that I’ve been involved in. Totally frustrating at times, and it reminded me of what a powerful tool DCM can be, but how it needs the right, the right sort of nurturing within an organisation. (EM70004)*

As most experts were DCM trainers, their trial experiences impacted the way they trained future mappers.

*… it’s been really helpful for me as a DCM trainer. It’s been really helpful to see how people implement DCM in practice. … to see the things that work, the things that maybe don’t work as well, the areas where we might need to adapt the training to make sure that people are, we have more focus on the areas that people find difficult. (EM70006)*

#### Experts’ ability to predict further DCM implementation

There was little consistency between expert ratings of mappers’ abilities and number of DCM cycles completed in each care home (see Figs. 2 & 3). While caution needs to be exercised in interpreting these ratings, given they were subjective and could be influenced by factors associated with the expert as well as the mappers (e.g. their ability to mentor mappers, their own standards for DCM completion) they offer insight into issues around the feasibility of predicting ongoing levels of support that might be needed to sustain implementation of DCM. Experts expressed concerns regarding mapper capabilities in half of the homes where two or three full cycles were completed and no concerns about four homes where only the first supported cycle was completed. The qualitative notes also supported this finding: experts expressed little or no concern about future use of DCM in seven homes, there of which only completed the first cycle and the others two or 3 cycles. One expert expressed significant concerns about a home where the mappers subsequently completed all 3 cycles. These data suggest that expert predictions of mappers’ ongoing support needs, after supporting them through one DCM cycle, are unlikely to be a good estimate of the degree of supported needed to complete further cycles. Due to the pragmatic trial design experts were only permitted to support homes in their first cycle and so were unable to provide proactive, ongoing support to homes, despite any concerns. Mappers were however able to access telephone or e-mail support from the trial’s DCM lead, for the remainder of the trial. These data suggest that expert predictions of mappers’ ongoing support needs, after supporting them through one DCM cycle, are unlikely to be a good estimate of the degree of supported needed to complete further cycles.

## Discussion

This study has identified that support from an external expert was essential to successful implementation of DCM by care home mappers. The benefits of external support for implementing interventions in care homes has been reported in other research [41] including evidence from previous research on DCM, which found support for new mappers by experienced in-house mappers facilitated implementation [31]. Experts and mappers indicated the 5 days of support provided were not sufficient to support a full cycle of DCM. While expert judgements were not found to be an accurate predictor of whether homes were likely to complete more than their first supported cycle, expert data collection forms, showed mappers in around half of the care homes were rated as unable to use at least one DCM component accurately in the future. Just under two thirds of homes were judged to need further support for future cycles. These findings indicate that attending DCM training plus completing one expert supported cycle is unlikely to provide enough training and support for sustainable care home staff led DCM implementation in most care homes. The provision of further ongoing support would potentially offer greater chance of sustaining DCM implementation beyond the first cycle. While recommended, [20] support for new mappers by an experienced mapper, is not currently a standard component of DCM, unless the organisation already has experienced mappers or purchases additional expert support. The findings of this study suggest consideration should be given to incorporating external support for mappers working in care homes that have no access to existing experienced mappers. The degree of support required is, this study suggests, likely to be resource intensive and thus creates significant cost implications. These need to be considered alongside the potential benefits that might be achieved through effective and sustained DCM implementation. Alternatively, this data raises the question about DCM’s suitability as a care home staff-led psychosocial intervention.

The expert support provided in this trial included increasing accuracy in use of all elements of the DCM process, building mappers’ confidence and keeping the process on track through prompting cycle components to be completed. While many mappers appreciated this, it was felt to create undue pressure by others. Experts who did not know the residents providing advice to staff about care practices, also created tensions within some homes. Building positive relationships between experts and care home mappers, and being flexible and understanding were viewed positively by care home staff. This is consistent with findings from previous research examining external expert [16] and staff [17] perceptions of external expert support for intervention implementation in care homes, which indicated experts’ understanding of a home’s needs and building positive relationships with staff are key to care home engagement, alongside flexibility of delivery if the intervention implementation to be sustained. This need for intervention flexibility and practicability was also identified as important in a study evaluating the impact of a national network of research ready care homes in England [42]. Care home managers reported that when deciding if to participate in research they considered whether the intervention would fit into the care home workload, practices and routines and the degree of control they would retain over implementation.

While other studies have adopted the expert role within interventions [16, 17, 43] the expert role is not a standard component of DCM. In this pragmatic trial the expert mapper’s role was to support the care home mappers to implement DCM. However, the degree of support offered and boundaries around defined support were sometimes unclear. Where mappers experienced implementation challenges related to broader contextual issues, such as unsupportive managers or care home cultures, there was sometimes an expectation that the expert would intervene. Experts had to judge what they could and could not do in such circumstances. There is, therefore, a careful line to be drawn between pragmatic levels of expert support for DCM within a pragmatic trial and support where the expert rather than care home mappers lead changes needed to make implementation possible. Previous research has indicated that successful care home interventions need to consider and address whole home contextual issues, such as the environment, culture and practices [44]. Given this and that DCM implementation beyond the first supported cycle was poor in this trial, further consideration should be given to if and how, whole home readiness for interventions are considered and how they are subsequently accommodated as part of standard intervention implementation.

This study also identified a range of challenges that mappers identified in relation to DCM as a tool and its implementation that were not able to be addressed even through expert support. These are reported in detail elsewhere [39]. For example difficulties in accessing and using computers to produce DCM reports and mappers lacking adequate understanding about person-centred care, DCM as a tool and how to apply it in practice following successful completion of training. This has implications for DCM as a tool and for the contents of the standard DCM training course with regards to how well it is able to prepare individuals to implement DCM sustainably.

A further, but additional outcome of external expert support identified in other studies, are the benefits that occur in terms of wider expert knowledge gains [17, 45]. In this study the expert mappers discussed how working with the care homes had enhanced their own knowledge and skills around DCM implementation and how they subsequently had applied this into work with other trial homes, but also to inform the way they trained future mappers. Thus, while resource intensive, use of this role did serve to enhance the way DCM training is delivered and how mappers are prepared to undertake this complex role.

In terms of intervention research in long-term care more, this study indicates that external expert support is a potentially a vital implementation component and should be considered as part of the provided resources. Where interventions are complex and are led by care home staff, extensive, ongoing support from an external expert may be required in order to support embedding of the intervention into everyday practice. This has significant resource implications, particularly in large studies where sites may be widely geographically spread and has particular implications for translation of effective interventions into routine practice.

### Strengths, limitations and future research

The role of expert support for care home intervention implementation is relatively under researched. This research adds to our understanding of the benefits and challenges associated with such a role. A strength of this study compared to previous published studies is the inclusion of multiple perspectives permitting agreements and contrasting opinions to be identified. Limitations of the study include the selection of 18 of the 31 intervention care homes for the interview component. While the homes were selected to reflect the range of full cycles completed, they may not be representative of all the intervention care homes. As process evaluation data was collected after completion of the final 16-month trial follow-up data, interviewees were asked to recall and reflect on the implementation process. This might generate different perceptions than if interviews had been conducted continually throughout the trial. We were also unable to collect interview data from experts, mappers and managers who had left post during the trial and thus their perspective is not captured.

Future intervention research in care homes should consider if and how external expert support can be embedded within the intervention, to ensure it does not become an unintended active intervention component. Since expert time is costly, future research should also consider the nature and amount of expert support required to successfully implement and sustain an intervention. To be cost effective, this may require tailored support that is based on care home needs and which may vary according to a range of contextual factors.

## Conclusions

This study suggests that ongoing support from an external expert may form a key component for successful staff led implementation of interventions in care homes. External support for a single cycle was not sufficient to facilitate ongoing implementation and therefore should be considered on a longer term basis. While this may be resource intensive, this needs to be balanced against the risk of partial or failed implementation without such support. Tools to support experts to be able to better identify areas of concern and to predict likelihood of mappers and care homes being able to sustainably use DCM independently would be imperative to appropriate resource use. Future research should consider how external support can effectively be built into care home interventions and ways to assess support whether tailored levels of support could be offered.

## Data Availability

The datasets used and/or analysed during the current study are available from the corresponding author on reasonable request.

## Appendix

### Topic guides – DCM EPIC Trial

***Topic Guide for Interviews with Care Home Manager***

- How have you found using DCM in your home?

*Could probe about stages of the process:*

*DCM Training (identifying & retaining mappers, releasing staff for training).*

*Briefing sessions.*

*Mapping & report writing.*

*Feedback sessions.*

*Action planning & making changes to practice*

- What worked well?
  - *Were any parts of the process particularly beneficial?*
- What were the challenges?
  - *How did you try to overcome these?*
  - *What strategies were most helpful?*
- What impact, if any, has the DCM project had?
  - *On residents/ care*
  - *On staff?*
  - *Any unexpected or wider impacts? (*e.g. *to other homes, care home trust)*
  - *Have those impacts been maintained? (if yes, how?)*

*(If no impacts are reported explore why not)*

- Would anything need to change for DCM to work successfully in your home?

***Topic Guide for Interviews with Mappers***

- How have you found using DCM in your home?

*Could probe about each stage:*

- *DCM Training (& being identified as a mapper)*
- *Briefing sessions*
- *Mapping & report writing*
- *Support from the expert mappers (Did it help? Could it have been improved?)*
- *Feedback sessions & sharing results (How shared & receptiveness of staff)*
- *Action planning & making changes to practice*

- What worked well?
  - *Were any parts of the process particularly beneficial?*
- What were the challenges?
  - *How did you try to overcome these?*

- *What strategies were most helpful?*

- What impact, if any, has the DCM project had?
  - *On residents/care?*
  - *On staff?*
  - *Any unexpected or wider impacts? (*e.g. *other homes, care home trust)*
  - *Have those impacts been maintained? (if yes, how?)*

*(If no impacts are reported explore why not)*

- Would anything need to change for DCM to work successfully in your home?

## Abbreviations

CH: Care Home
DCM: Dementia Care Mapping
EM: Expert Mapper
